# Supplementary material for: Distinct genetic architectures and environmental factors associate with host response to the γ2-herpesvirus infections
Source: Nat Commun. 2020 Jul 31;11:3849. doi: 10.1038/s41467-020-17696-2 (PMC7395761; doi:10.1038/s41467-020-17696-2)
Supplement: Supplementary file 1 — Supplementary Information [file 41467_2020_17696_MOESM1_ESM.pdf]

Distinct genetic architectures and environmental factors associate with host response to the γ2-herpesvirus infections.

Sallah et al.

Supplementary Tables

Supplementary Table 1. Seroprevalence of co-infection with EBV or KSHV

Supplementary Table 2. Associations with previously identified candidate variants

Supplementary Table 3. Loci with significant evidence of association with anti-EBV IgG levels (N=3,289)

Supplementary Table 4. Credible set showing strong evidence of association with anti-EBNA-1 IgG levels after meta-analysis

Supplementary Figures

Supplementary Figure 1. Burden of viral infections tested in the GPC Round 22 (2011).

Supplementary Figure 2. Correlation matrix of antibody responses (MFI) for KSHV and EBV

Supplementary Figure 3. Genome-wide association QQ-plots of all IgG response levels in 4,365 individuals

Supplementary Figure 4. Genome-wide association results of anti-KSHV IgG response levels in 4,365 individuals

Supplementary Figure 5. Genome-wide association results of anti-EBV IgG response levels in 4,365 individuals

Supplementary Figure 6. Trans-ethnic meta-analysis association plot for EBNA-1 IgG response levels in 6152 individuals of Ugandan and European Ancestry (EUR)

Supplementary Tables

Supplementary Table 1. Seroprevalence of co-infection with EBV or KSHV

Infection	EBV (N=3956)	KSHV (N=3988)
EBV	-	3800 (95%)
KSHV	3800 (96%)	-
HIV	267 (6.7%)	179 (4.5%)
HBV	118 (2.9%)	101 (2.5%)
HCV	151 (3.8%)	124 (3.1%)

N represents the number of seropositive individuals

Supplementary Table 2. Associations with previously identified candidate variants

Gene	Variant	P_1 (OR)	$P_{UG.ORF73}$ (b)	$P_{UG.K10.5}$ (b)	$P_{UG.K8.1}$ (b)
IL12A	rs568408	0.02 (2.4)	0.006 (-0.08)	0.81 (-0.006)	0.18 (-0.04)
IL6	rs1800795	0.04 (N.R)	0.42 (-0.08)	0.91 (0.01)	0.58 (-0.05)
IL4	rs2243248	0.05 (2.8)	0.77 (0.01)	0.96 (0.001)	0.64 (0.01)
IL13	rs20541	0.01 (1.88)	0.87 (-0.004)	0.56 (-0.01)	0.62 (-0.01)
IL8RB	rs1126579	0.003(0.49)	0.87(0.01)	0.36 (0.03)	0.27(0.04)
IL6	rs1800795	0.05 (3.7)	0.42 (-0.07)	0.91 (0.01)	0.58 (0.05)
FCgRIIIA	rs396991	0.00028 (N.R)	0.31 (-0.03)	0.93 (-0.003)	0.67 (-0.01)
IRAK1	rs1059702	N.R	0.37 (0.05)	0.90 (0.05)	0.77 (0.02)
HLA-A	*A*6801*	0.02 (2.6)	0.38(-0.09)	0.37(-0.09)	0.08(-0.19)
HLA-A	*A*30*	0.019 (0.48)	0.22(-0.03)	0.21(-0.03)	0.62(-0.01)
HLA-B	*B*14*	0.033 (4.27)*	0.76(0.01)	0.85(-0.01)	0.83(-0.01)
HLA-B	*B*58*	0.00001 (0.03)	0.88(0.0005)	0.40(0.03)	0.49(-0.02)
HLA-C	*C*0701*	0.002 (1.6)	0.39(0.03)	0.16(0.04)	0.42(0.03)
HLA-C	*C*05*	0.0006 (0.32)	0.19(0.14)	0.15(0.16)	0.62(0.06)
HLA-C	*C*07*	0.01 (2.4)	0.92(-0.003)	0.68(0.01)	0.91(0.003)
HLA-DQB1	*DQB1*0502*	0.0465 (0.519)	0.29(0.14)	0.83(-0.03)	0.56(0.08)
HLA-DQB1	*DQB1*0604*	0.0017 (7.74)	0.90(-0.005)	0.73(0.01)	0.95(-0.002)
HLA-DRB1	*DRB1*04*	0.02 (3.4)	0.78(-0.02)	0.24(0.09)	0.90(0.0008)
HLA-DRB1	*DRB1*1104*	0.0473 (2.1)	0.19(0.17)	0.94(-0.009)	0.49(0.09)
HLA-DRB1	*DRB1*1302*	0.0037 (5.82)	0.82(0.008)	0.59(-0.02)	0.65(0.01)

P_1 – P-value from original study

$P_{UG.ORF73}$– P-value from Uganda GPC anti-ORF73 IgG GWAS

$P_{UG.K8.1}$ – P-value from Uganda GPC anti-K8.1 IgG GWAS

$P_{UG.K10.5}$ – P-value from Uganda GPC anti-K10.5 IgG GWAS

N.R – Not reported

*FDR (False Discovery Rate)

Supplementary Table 3. Loci with significant evidence of association with anti-EBV IgG levels (N=3,289)

Trait	Chr:Pos(b37)	Variant	Nearest Gene[a]	Consequence[a]	EA	EAF (%)	P	b (95% C.I)
EBNA-1	6:32604654	rs9272371	HLA-DQA1	Intron	C	26.9	$5.24x10^{-33}$	-0.37 (-0.43 – -0.31)
EBNA-1	6:32404220	rs3129867	HLA-DRA	Upstream	G	49.2	$1.63x10^{-22*}$	-0.25(-0.30 - -0.20)
VCA	6: 32615738	rs28394498	HLA-DQA1	Intron	T	17.9	$9.15x10^{-11}$	-0.23 (-.29 - -0.16)

EA – Effect Allele, EAF – Effect Allele Frequency

[a]Annotated using Ensembl VEP

*P.conditional on lead SNP, b (95%C.I)=6.01 $x10^{-11}$, -0.17(-0.22 - -0.12)

Supplementary Table 4. Credible set showing strong evidence of association with anti-EBNA-1 IgG levels after meta-analysis

			Alleles	Ugandan (N=4365)				European Ancestry (N=2162)				MANTRA EUR + UG (N=6527)	
Lead SNP	Chr:Pos(b37)	Locus	Effect/ Other	EAF	Beta	SE	P	EAF	Beta	SE	P	$log_{10}BF$	P_Q*
rs6927022[a]	6:32612397	HLA-DRB1	A/G	0.73	0.26	0.02	$1.36x10^{-21}$	0.59	0.16	0.015	$7.35x10^{-26}$	44.3	0.06
rs9272371[b]	6:32604654	HLA-DQA1	C/T	0.3	-0.36	0.02	$3.63x10^{-44}$	0.37	-0.02	0.015	0.14	42.6	$3.56x10^{-8}$
rs9274247	6:32631295	HLA-DQB1	A/G	0.22	-0.32	0.02	$2.63x10^{-29}$	0.35	-0.13	0.015	$4.51x10^{-16}$	42.2	0.63

EAF - Effect Allele Frequency

SE – Standard Error

[a]European (EUR) lead SNP

[b]Ugandan (UG) lead SNP

*P_Q – Cochran's Q-test for heterogeneity

Supplementary Figures

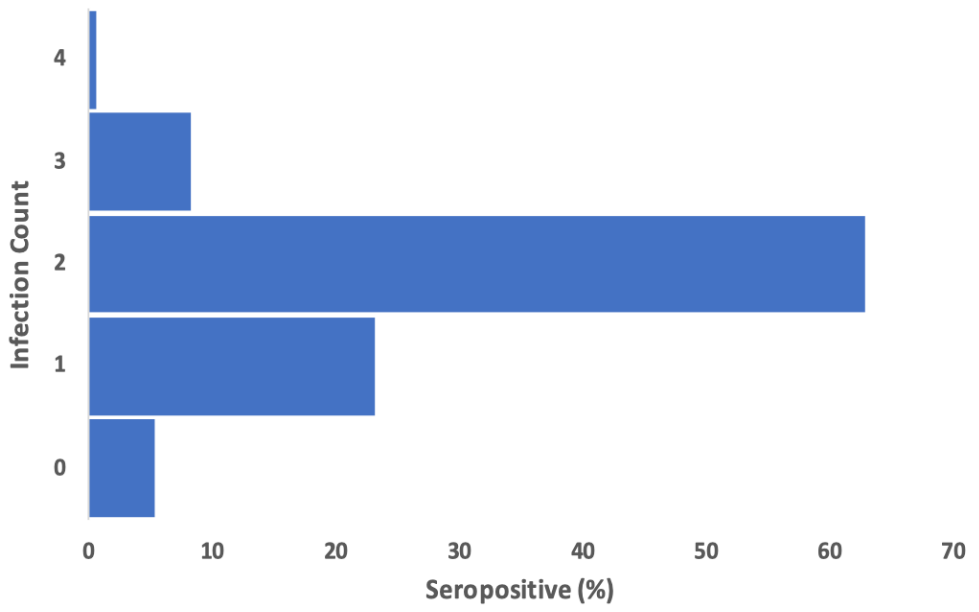

Supplementary Figure 1. Burden of viral infections tested in the GPC Round 22 (2011). The number of seropositive reactions to viruses for all participants, the infection count represents the minimum number of infections participants are seropositive for.

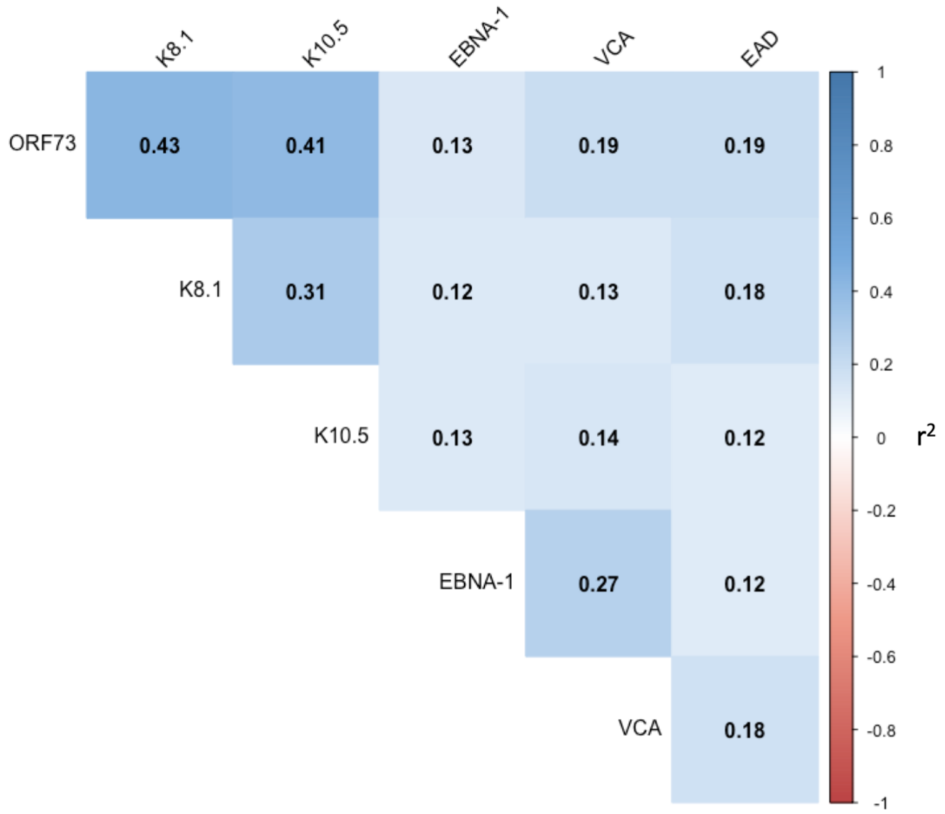

Supplementary Figure 2. Correlation matrix of antibody responses (MFI) for KSHV and EBV. Positive correlations are in blue and intensity is proportional to the correlation coefficients (r^2) labelled in the squares and indicated on the right-hand side of the correlogram. All tests meet Pearson's significance threshold of $p<0.05$.

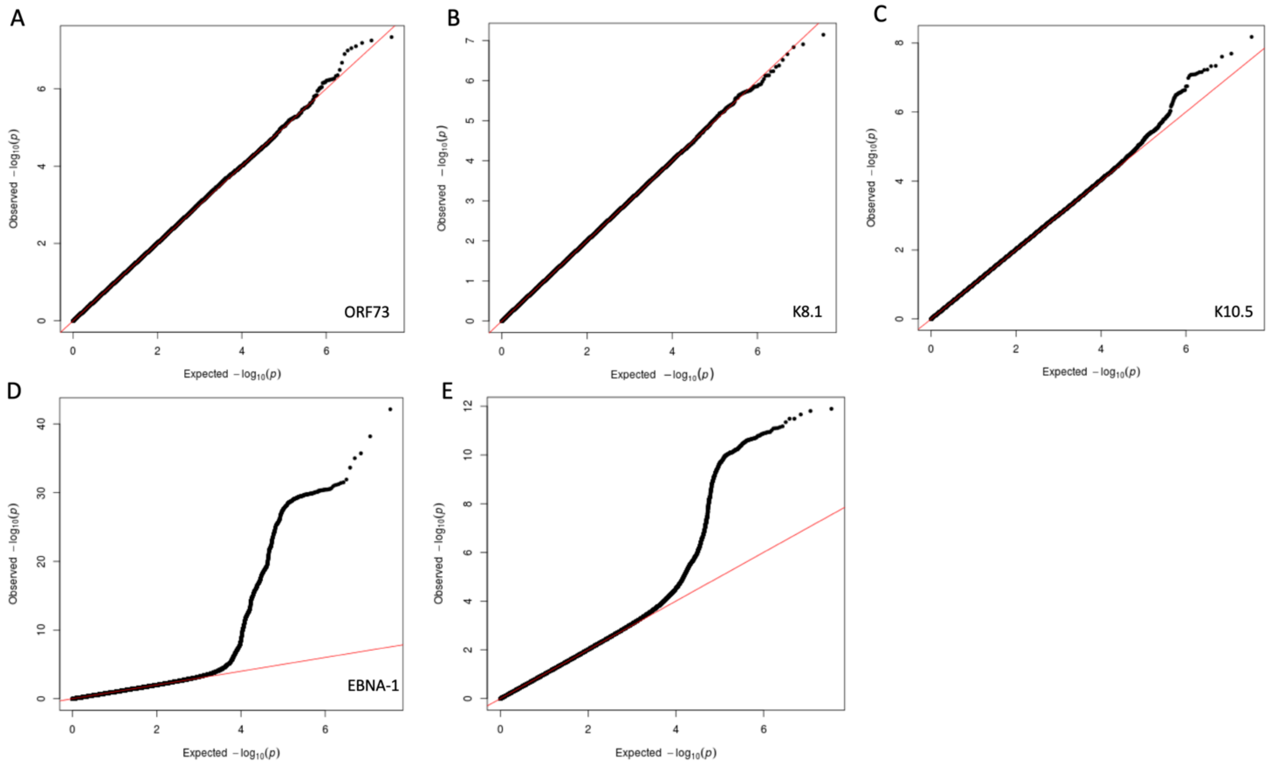

Supplementary Figure 3. Genome-wide association QQ-plots of all IgG response levels in 4,365 individuals. A. Anti-Orf73 IgG association. B. Anti-K8.1 IgG association. C. Anti-K10.5 IgG association. D. Anti-EBNA-1 IgG association. E. Anti-VCA IgG association.

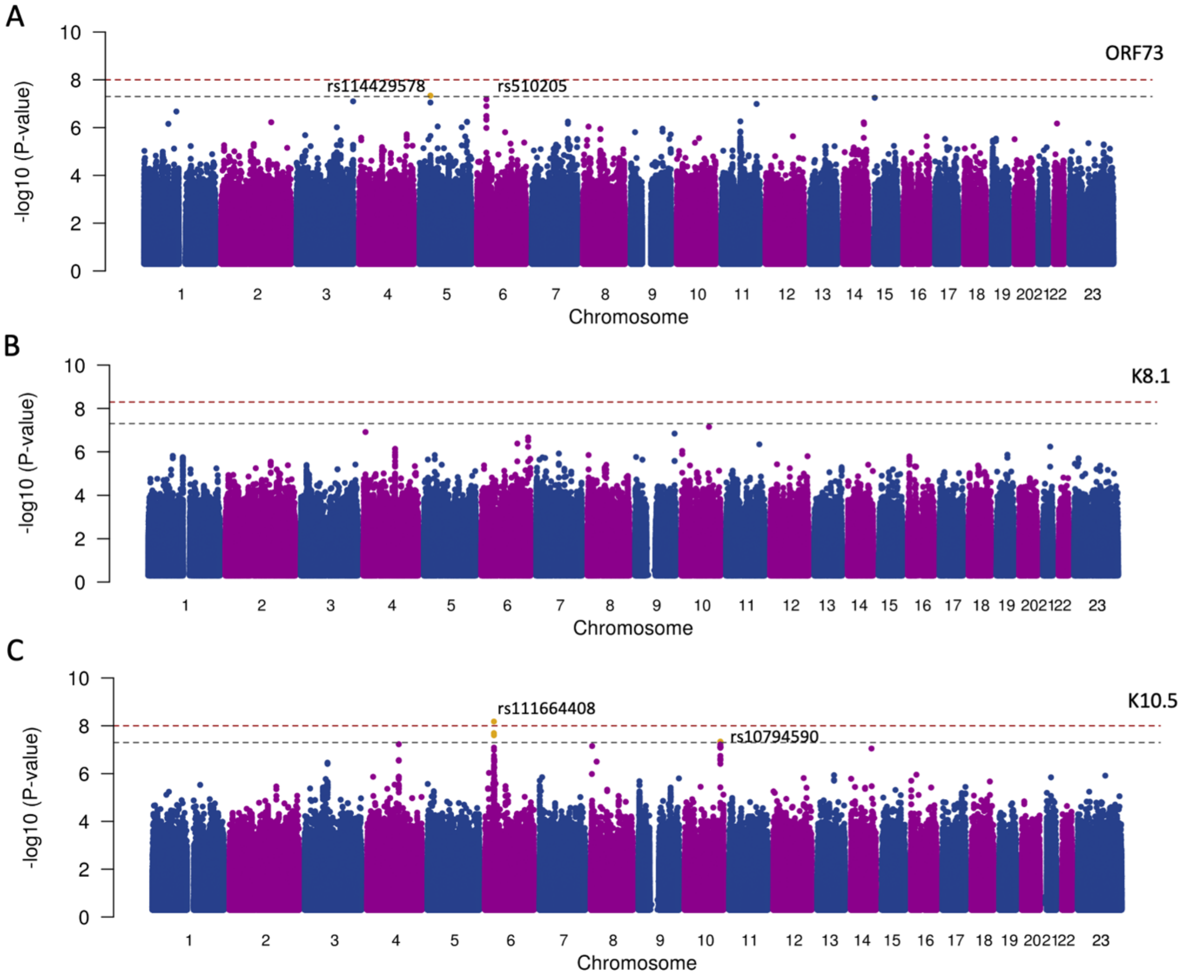

Supplementary Figure 4. Genome-wide association results of anti-KSHV IgG response levels in 4,365 individuals. A. Anti-ORF73 IgG association. B. Anti-K8.1 IgG association. C. Anti-K10.5 IgG association. GWAS performed using linear mixed model accounting for kinship in GEMMA. Red dashed line: Genome wide significance threshold ($p<1x10^{-8}$), grey dashed line: Standard genome-wide significance threshold ($p<5x10^{-8}$), 23=X Chromosome. Yellow: SNPs that meet the standard genome-wide significance threshold ($p<5x10^{-8}$).

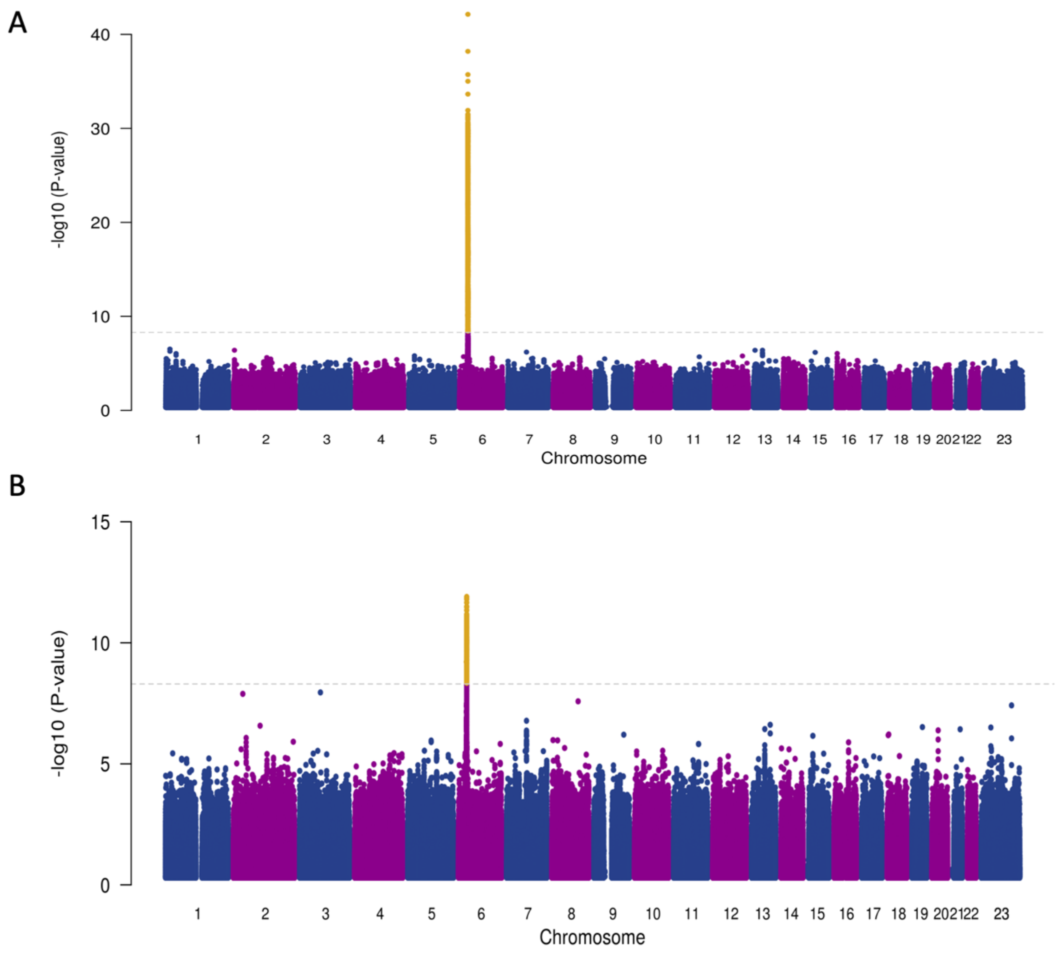

Supplementary Figure 5. Genome-wide association results of anti-EBV IgG response levels in 4,365 individuals. A. Anti-EBNA-1 IgG association. B. Anti-VCA IgG association. Manhattan Plot: Grey dashed line: Genome wide significance threshold ($p<1x10^{-8}$), 23=X Chromosome. Yellow: SNPs that meet the genome-wide significance threshold.

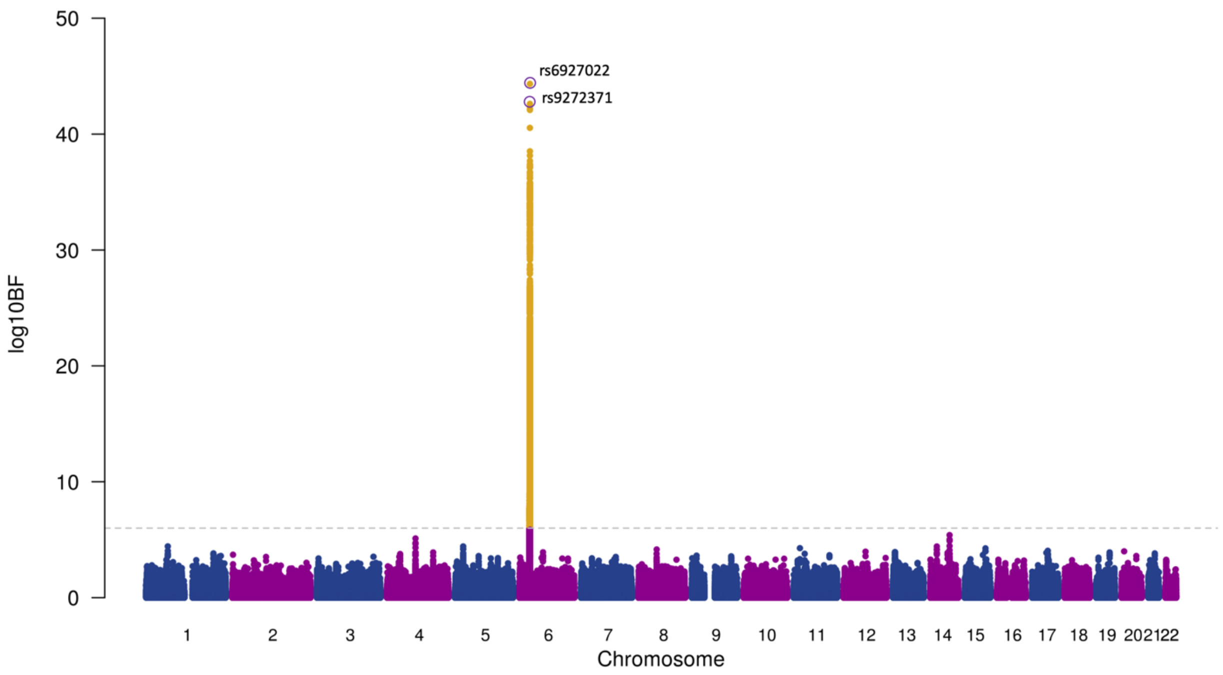

Supplementary Figure 6. Trans-ethnic meta-analysis association plot for EBNA-1 IgG response levels in 6152 individuals of Ugandan and European Ancestry (EUR). The lead SNPs for EUR (rs6927022, log10BF= 44.3) and Uganda (rs9272371, log10BF= 42.6) GWASs on chromosome 6 within the *HLA* region are labelled and circled in purple. Grey dashed line: threshold= $\log_{10}$BF > 6. Yellow: SNPs that meet the statistical significance threshold.
