# Supplementary material for: Distinct genetic architectures and environmental factors associate with host response to the γ2-herpesvirus infections
Source: Nat Commun. 2020 Jul 31;11:3849. doi: 10.1038/s41467-020-17696-2 (PMC7395761; doi:10.1038/s41467-020-17696-2)
Supplement: Supplementary file 3 — Descriptions of Additional Supplementary Files [file 41467_2020_17696_MOESM3_ESM.docx]

**Descriptions of Additional Supplementary Files**

**Supplementary Data 1**

**Description:** Predictors of KSHV antibody response

**Supplementary Data 2**

**Description:** Predictors of EBV IgG antibody response

**Supplementary Data 3**

**Description:** Summary Association Results for KSHV IgG antibody levels and Imputed Classical FourDigit HLA- Alleles

**Supplementary Data 4**

**Description:** Summary Association Results for EBV IgG antibody levels and Imputed Classical FourDigit HLA Alleles

**Supplementary Data 5**

**Description:** Pairwise Linkage Disequilibrium (r2 Values) between HLA--DRB1:15*01 and Imputed Classical HLA- Alleles
