# Supplementary material for: Distinct genetic architectures and environmental factors associate with host response to the γ2-herpesvirus infections
Source: Nat Commun. 2020 Jul 31;11:3849. doi: 10.1038/s41467-020-17696-2 (PMC7395761; doi:10.1038/s41467-020-17696-2)
Supplement: Supplementary file 9 — Reporting Summary [file 41467_2020_17696_MOESM9_ESM.pdf]

nature research

Corresponding author(s): Neneh Sallah, Inês Barroso

Last updated by author(s): Jul 1, 2020

Reporting Summary

Nature Research wishes to improve the reproducibility of the work that we publish. This form provides structure for consistency and transparency in reporting. For further information on Nature Research policies, see Authors & Referees and the Editorial Policy Checklist .

Statistics

For all statistical analyses, confirm that the following items are present in the figure legend, table legend, main text, or Methods section.

n/a	Confirmed	
☐	☒	The exact sample size (*n*) for each experimental group/condition, given as a discrete number and unit of measurement
☐	☒	A statement on whether measurements were taken from distinct samples or whether the same sample was measured repeatedly
☐	☒	The statistical test(s) used AND whether they are one- or two-sided<br>*Only common tests should be described solely by name; describe more complex techniques in the Methods section.*
☐	☒	A description of all covariates tested
☐	☒	A description of any assumptions or corrections, such as tests of normality and adjustment for multiple comparisons
☐	☒	A full description of the statistical parameters including central tendency (e.g. means) or other basic estimates (e.g. regression coefficient) AND variation (e.g. standard deviation) or associated estimates of uncertainty (e.g. confidence intervals)
☐	☒	For null hypothesis testing, the test statistic (e.g. *F*, *t*, *r*) with confidence intervals, effect sizes, degrees of freedom and *P* value noted<br>*Give P values as exact values whenever suitable.*
☒	☐	For Bayesian analysis, information on the choice of priors and Markov chain Monte Carlo settings
☒	☐	For hierarchical and complex designs, identification of the appropriate level for tests and full reporting of outcomes
☐	☒	Estimates of effect sizes (e.g. Cohen's *d*, Pearson's *r*), indicating how they were calculated

Our web collection on statistics for biologists contains articles on many of the points above.

Software and code

Policy information about availability of computer code

Data collection	No software used
Data analysis	R Version 3.4<br>PLINK 2.0<br>IMPUTE2<br>FaST-LMM<br>GEMMA<br>QUANTO

For manuscripts utilizing custom algorithms or software that are central to the research but not yet described in published literature, software must be made available to editors/reviewers. We strongly encourage code deposition in a community repository (e.g. GitHub). See the Nature Research guidelines for submitting code & software for further information.

Data

Policy information about availability of data

All manuscripts must include a data availability statement. This statement should provide the following information, where applicable:

- Accession codes, unique identifiers, or web links for publicly available datasets
- A list of figures that have associated raw data
- A description of any restrictions on data availability

Summary statistics are available on the NHGRI-EBI GWAS Catalog https://www.ebi.ac.uk/gwas/downloads/summary-statistics under accession codes: GCST90000522, GCST90000523, GCST90000524, GCST90000525, GCST90000526.

Field-specific reporting

Please select the one below that is the best fit for your research. If you are not sure, read the appropriate sections before making your selection.

- [x] Life sciences
- [ ] Behavioural & social sciences
- [ ] Ecological, evolutionary & environmental sciences

For a reference copy of the document with all sections, see nature.com/documents/nr-reporting-summary-flat.pdf

Life sciences study design

All studies must disclose on these points even when the disclosure is negative.

Sample size	The statistical power to identify genetic variants of genome-wide significance and with different effect sizes given the sample size was estimated using QUANTO software (http://biostats.usc.edu/software).
Data exclusions	Stringent variant and sample quality control (QC) filtering was performed. Low quality variants that mapped to multiple regions within the human genome or did not map to any region, and duplicate variants genotyped on the chip were removed. We excluded samples with a call rate <97% and heterozygosity >3 SD from the mean, discordant genetic sex and reported sex, and sites deviating from Hardy Weinberg equilibrium (p<10-8). Following imputation, we only included high quality sites (info score >0.3 and r2>0.6) with minor allele frequency (MAF) >= 0.5%. We also removed samples without matching phenotype and genotype or sequence data.
Replication	For GWAS, we used a stringent p-value threshold of 1x10-8 accounting for an FDR of 5% and low LD in population. We have also replicated findings from previous studies.
Randomization	NA observational study
Blinding	NA observational study

Reporting for specific materials, systems and methods

We require information from authors about some types of materials, experimental systems and methods used in many studies. Here, indicate whether each material, system or method listed is relevant to your study. If you are not sure if a list item applies to your research, read the appropriate section before selecting a response.

Materials & experimental systems

n/a	Involved in the study	
[ ]	[x]	Antibodies
[x]	[ ]	Eukaryotic cell lines
[x]	[ ]	Palaeontology
[x]	[ ]	Animals and other organisms
[ ]	[x]	Human research participants
[x]	[ ]	Clinical data

Methods

n/a	Involved in the study	
[x]	[ ]	ChIP-seq
[x]	[ ]	Flow cytometry
[x]	[ ]	MRI-based neuroimaging

Antibodies

Antibodies used	goat F(Ab')2 Anti-Human IgG (γ), R-PE conjugate, (Life Technologies, Grand Island, NY now Thermo Fisher)
Validation	https://www.thermofisher.com/antibody/product/Goat-anti-Human-IgG-Fc-Secondary-Antibody-Polyclonal/H10104

Human research participants

Policy information about studies involving human research participants

Population characteristics	source population: open population-based cohort of ~25000 inhabitants of 25 villages in rural southwest Uganda of numerous ethno-linguistic groups, prevalently Baganda. HIV prevalence is stable at ~7%. See methods and https://www.ncbi.nlm.nih.gov/pmc/articles/PMC3600628/
Recruitment	during annual visits in ongoing prospective study with total enumeration of the population, 7000 individuals >13 years of age and both sexes were randomly selected, representing 11 ethnicities. See above
Ethics oversight	Uganda Virus Research Institute Science and Ethics Committee following Uganda National Council of Science and Technology (UNCST) guidelines

Note that full information on the approval of the study protocol must also be provided in the manuscript.
